# Hemoglobin H Disease in Turkey: Experience from Eight Centers

**DOI:** 10.4274/tjh.2014.0461

**Published:** 2016-02-17

**Authors:** Selma Ünal, Gönül Oktay, Can Acıpayam, Gül İlhan, Edip Gali, Tiraje Celkan, Ali Bay, Barış Malbora, Nejat Akar, Yeşim Oymak, Tayfur Toptaş

**Affiliations:** 1 Mersin University Faculty of Medicine, Department of Pediatric Hematology, Mersin, Turkey; 2 Antakya Hemoglobinopathy Diagnosis, Control, and Education Center, Antakya, Turkey; 3 Antakya Education and Research Hospital, Antakya, Turkey; 4 İstanbul University Cerrahpaşa Faculty of Medicine, Department of Pediatric Hematology, İstanbul, Turkey; 5 Gaziantep University Faculty of Medicine, Department of Pediatric Hematology, Gaziantep, Turkey; 6 Dr. Sami Ulus Education and Research Hospital, Ankara, Turkey; 7 TOBB University Faculty of Medicine, Department of Pediatric Hematology, Ankara, Turkey; 8 Behçet Uz Education and Research Hospital, İzmir, Turkey; 9 Van Education and Research Hospital, Van, Turkey

**Keywords:** Thalassemia, Hemoglobinopathy, Hemoglobin H disease

## Abstract

The purpose of this study was to research the problem of hemoglobin H (HbH) disease, to reveal the distribution patterns among different health centers, and to emphasize the importance of this disease for Turkey. A total of 273 patients were included from 8 hemoglobinopathy centers. The Antakya Hemoglobinopathy Center reported 232 patients and the remaining 7 centers reported 41 patients. PubMed was also searched for published articles related to Turkish patients with HbH disease, and we found 16 articles involving a total of 198 HbH patients. Most of the patients were reported from Antakya; thus, special attention should be paid to this region. This is a preliminary study to investigate the extent of the problem of HbH disease and it emphasizes the need for hematology associations or the Ministry of Health to record all cases of HbH disease in Turkey.

## Abstract (tr)

Bu çalışmada, ülkemizdeki hemoglobin H (HbH) hastalığı sorununu irdelemek, bölgelere göre dağılımını tespit etmek ve sonuç olarak HbH hastalığının ülkemiz için önemini vurgulamak amaçlandı. Sekiz hemoglobinopati merkezinden toplam 273 hasta çalışmaya dahil edildi. Merkezler arasında en fazla bildirimi 232 hasta ile Antakya Hemoglobinopati Merkezi sağladı. Kalan 7 merkezden toplam 41 HbH hastası bildirildi. Eş zamanlı olarak PubMed’den Türkiye’den yayınlanan ya da Türk HbH hastalarının dahil edildiği makaleler araştırıldı ve sonuçta 198 HbH hastasını içeren toplam 16 yayına ulaşıldı. Bu çalışma, ülkemizde HbH hastalığı sorununu araştıran bir ön çalışmadır. Çalışmamıza Antakya’dan oldukça fazla hasta bildiriminin olması HbH hastalığı yönünden dikkatimizi bu bölgeye yöneltmemize neden olacaktır. Sağlık Bakanlığı önderliğinde oluşturulacak kayıt sistemleri ile tüm HbH’li hastalar kayıt altına alınmalı ve böylece HbH hastalığının ülkemiz için önemi vurgulanmalıdır.

## INTRODUCTION

Hemoglobin H (HbH) disease occurs due to defects in 3 of the 4 alpha genes found in healthy people. HbH (--/-α) is compatible with life and usually has a similar presentation to that of thalassemia intermedia [1]. However, clinical signs vary among patients, and while some patients may need intermittent or frequent transfusions, others do not.

HbH disease is suspected in cases of unresponsiveness to iron replacement therapy and findings of microcytic anemia in complete blood counts and peripheral smears. In the early decades of the disease, most patients do not need erythrocyte transfusions. Diagnosis is established when 5%-30% HbH is detected on Hb electrophoresis. Patients with HbH disease also have 20%-40% Hb Barts in the evaluation of cord blood [2]. Brilliant cresyl staining can be used as a screening procedure where the molecular diagnosis of HbH disease is not possible [3].

The prevalence of alpha thalassemia is 0.24% worldwide and 13,000 babies with HbH are born annually [4]. The overall incidence rate of alpha thalassemia for Turkey is reported to be 0.25%-4.1% [5,6,7]. However, according to different single-center studies from the south of Turkey, the frequency of alpha thalassemia ranges between 2.5% and 7.5% [4,5,6,7,8,9,10,11,12].

As the incidence of alpha thalassemia is high, the prevalence of HbH disease may be assumed to be higher. However, due to inadequate reporting of patients with HbH disease, the exact spread and occurrence rates of HbH disease cannot be determined. Thus, the patients who were reported from eight different centers and those reported from previously published studies related to Turkish cases of HbH disease were included in the present study.

## MATERIALS AND METHODS

Hemoglobinopathy centers in Turkey were informed about and invited to participate in this study. Eight centers accepted the invitation and 273 patients were included in the study. Three of these centers were university hospitals and the others were state hospitals. The Antakya Hemoglobinopathy Diagnosis, Control, and Education Center (AHDCEC) reported 232 patients. The remaining 41 patients were reported from the other 7 centers. All of the data collected from these centers were evaluated retrospectively. Additionally, PubMed was searched for English publications related to Turkish patients with HbH disease. As publications in Turkish were difficult to locate and access, those sources were not included.

## RESULTS

Of the 273 patients included in the study, 125 were female and 148 were male. Their ages ranged between 9 month and 78 years (Table 1). Mean Hb level was 8.7 g/dL (range: 7.2-10.9), mean red blood cell count was 5.16 (x1012/L) (range: 4.07-5.8), mean MCV was 54.7 fL (range: 48-76.4), mean MCH value was 17.9 pg (range: 15-23.9), mean MCHC value was 32.2 g/dL (range: 28.4-34), mean RDW value was 26.7 (range: 14.6-28), mean HbA2 was 2% (range: 1.2%-2.8%), mean HbF was 2.9% (range: 0.2%-3.3%), and mean HbH was 4.7% (range: 1.8%-17.9%).

The AHDCEC reported 232 patients with HbH disease who were recorded in a data file reporting cases dating back to as far as 1993. In the evaluation of the patients from the AHDCEC, it was observed that the diagnosis of HbH disease was based on clinical findings, hemoglobin electrophoresis, and HbH detection, but alpha gene mutation analysis was not performed for any of these patients. The evaluation of data from 41 patients from the centers other than the AHDCEC revealed that only one patient reached adulthood with an age of 58. Premarital screening was performed in only one family and one other family had a history of hydrops fetalis. The centers contributing to the study and the numbers of patients from these centers are shown in Table 1.

The PubMed search for publications on Turkish patients with HbH disease yielded 16 articles with a total of 198 patients with HbH disease (Table 2) [10,13,14,15,16,17,18,19,20,21,22,23,24,25,26,27].

## DISCUSSION

Although reports about patients with beta thalassemia and sickle cell anemia are available, insufficient data exist about patients with HbH disease, which can be accompanied by various complications and moderate or severe anemia that may require transfusions.

A total of 273 patients from 8 centers were included in this study and 232 of these patients were from Antakya, which is located in the Mediterranean region and had high malaria incidence rates, probably due to Lake Amik, until recent years. It is known that alpha thalassemia is common where malaria is endemic. It has been observed that another reason why the majority of patients with HbH disease are reported from this area is that the doctors dealing with thalassemia have been working in the region for many years, and thalassemia patients have been recorded since 1993 at the AHDCEC. The low reported number of patients from cities within the same geographic region, such as Mersin and other centers of population, may be due to the recent start of recording patients with thalassemia and HbH disease.

We detected 198 patients with HbH disease when we searched articles from PubMed related to Turkish patients with HbH disease [10,13,14,15,16,17,18,19,20,21,22,23,24,25,26,27]. When the results of our study were added to that number, we found 471 HbH cases to date from Turkey, and this number is fairly high (Table 2). Since the origin of the patients was not recorded in other published studies, no other region was pointed out like Antakya where HbH disease was very frequent.

As the data used in this study were not obtained from every center in Turkey, this does not accurately reflect the real number of patients and data on HbH disease in Turkey. However, as many of the HbH patients were reported from Antakya, the situation of the disease in this particular region deserves attention first. In addition, it is necessary to design studies using the data from all centers in Turkey to determine the exact number of patients with HbH disease.

Another important point is that cases of hydrops fetalis due to alpha thalassemia are rarely reported in Turkey [28]. All cases should be reported and families with HbH should be evaluated for their history of nonimmunological hydrops fetalis. Prenatal diagnosis should be offered to families who have history of hydrops fetalis, in utero death, or abortion.

## CONCLUSION

There are no exact data related to the prevalence of HbH disease in Turkey. However, the 471 cases that are reported based on the data from the literature and the eight centers included in this study are noteworthy. Thalassemia is very common in Turkey, and there are several centers to follow the disease. Recording of HbH cases by these centers will illustrate the urgency of the thalassemia problem in Turkey.

In this study, it was found that Antakya is the region where HbH disease is most frequently encountered in Turkey. More studies are required to understand the facts about alpha thalassemia-HbH disease in Turkey.

## Figures and Tables

**Table 1:**
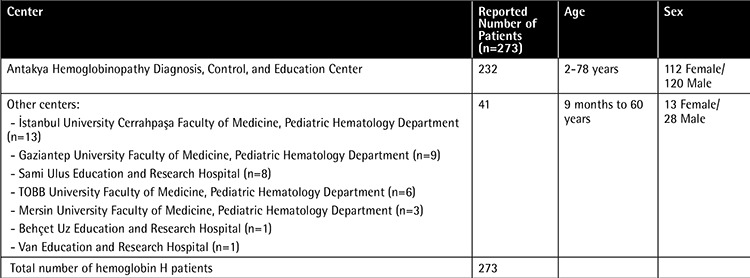
Centers involved in the study and number of hemoglobin H patients.

**Table 2:**
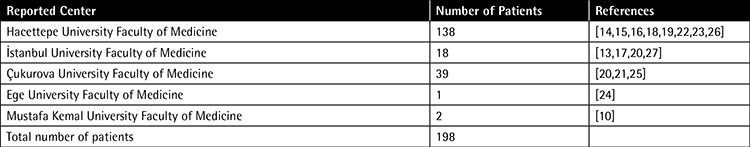
Articles associated with hemoglobin H disease in Turkey.
